# An unusual case of adamantinoma of long bone

**DOI:** 10.4322/acr.2021.276

**Published:** 2021-05-27

**Authors:** Arvind Kumar, Ruchi Sharma, Anil Kumar Verma, Abhijeet Tiwari, Jyoti Mishra

**Affiliations:** 1 All India Institute of Medical Sciences, Department of Pathology and Laboratory Medicine, Rishikesh, Uttarakhand, India; 2 All India Institute of Medical Sciences, Department of Transfusion Medicine & Blood Bank, Raipur, Chhattisgarh, India; 3 NH MMI Narayana Superspeciality Hospital, Raipur, India; 4 School of Medical Sciences and Research, Sharda Hospital, Department of Pathology, Greater Noida, Uttar Pradesh, India

**Keywords:** Adamantinoma, Diaphyses, Tibia

## Abstract

Adamantinoma of the long bones is an exceedingly rare and slow-growing tumor that affects the diaphysis of long bones, particularly the tibia. Based on the pattern of the epithelial cell component and the presence or absence of the osteofibrous dysplasia-like element, several histological variants have been described, such as (i) tubular (the most frequent), (ii) basaloid, (iii) squamous, (iv) spindle variant, (v) osteofibrous dysplasia –like variant, and (vi) Ewing’s sarcoma – like adamantinoma (the least frequent). The diagnosis may be challenging since this tumor may be mistakenly interpreted as carcinoma, myoepithelial tumor, osteofibrous dysplasia, and vascular tumor. We report the case of a 41-year-old male who presented with swelling over the right leg associated with pain. The X-ray showed a lytic lesion of the right-sided tibia. The diagnosis of adamantinoma was made based on the clinico-radiological, histomorphology, and immunohistochemical findings. Histologically, classic adamantinoma is a biphasic tumor characterized by epithelial and osteofibrous components in varying proportions and differentiating patterns. The diagnosis can be confirmed by immunohistochemistry for demonstrating sparse epithelial cell nests when the radiological features are strongly consistent with adamantinoma. This case is highlighted because the epithelial component can lead to a misdiagnosis, particularly when the clinico-radiological features are overlooked. Adamantinoma of long bones has the potential for local recurrence and may metastasize to the lungs, lymph nodes, or other bones. The prognosis is good if early intervention is taken.

## INTRODUCTION

Adamantinoma is a rare neoplasm that tends to involve the tibia almost exclusively. It is a primary low-grade, malignant bone tumor; of unknown histogenesis. The term “adamantinoma” comes from the histological resemblance of the neoplasm to ameloblastomas of the jaw. Other bones can also be involved, like the femur, ulna, humerus, and radius. The neoplasm may start as a fibular lesion with subsequent involvement of the ipsilateral tibia. Males and females are affected almost equally, and most patients are young adults.^1^ Because of the indolent nature of this malignancy, it typically has a long and progressive clinical course, which is initially characterized by swelling, pain, and deformity. The histogenesis of adamantinoma is still unclear, although several studies confirmed an epithelial differentiation.^2^ ^-^ ^4^ Adamantinoma comprises 0.1-0.5% of all primary bone tumors.^5^ A local recurrence and distant metastases have been described to occur many years after the primary lesion.^6^ ^-^ ^8^Cutaneous metastasis is also seen in some instances.^9^ Radiographically, the adamantinoma presents as a lytic, eccentric, cortical lesion involving the tibia’s shaft. The lesion often had a ‘bubbly’ appearance, and most of them show perilesional sclerosis. Soft tissue involvement is also seen in larger tumor cases. The radiological and histological features are often similar to osteofibrous dysplasia. However, as adamantinoma is a locally aggressive lesion, its differentiation from benign conditions is important. The magnetic resonance imaging (MRI) may help to make the diagnosis.^10^ Osteofibrous dysplasia and adamantinoma appear to be related, and osteofibrous dysplasia may be a precursor of adamantinoma.^11^ Grossly, adamantinoma is poorly defined and may extend into the overlying soft tissue.

Morphologically many patterns of growth have been described like (i) basaloid, (ii)spindle, (iii) squamoid, and (iv) tubular. The most common variant is the basaloid, which consists of solid nests of basaloid cells with palisading at the periphery.^12^ ^,^ ^13^ The electron microscopy and immunohistochemistry confirmed the epithelial nature of the tumor cells. The keratins expressed by adamantinoma are mainly CK14 and CK19. In contrast to other bone and soft tissue tumors with epithelial phenotypes such as synovial sarcoma, chordoma, and epithelioid sarcoma, the adamantinoma lacks immunoreactivity for CK8 and CK18.

## CASE REPORT

**Figure 1:**
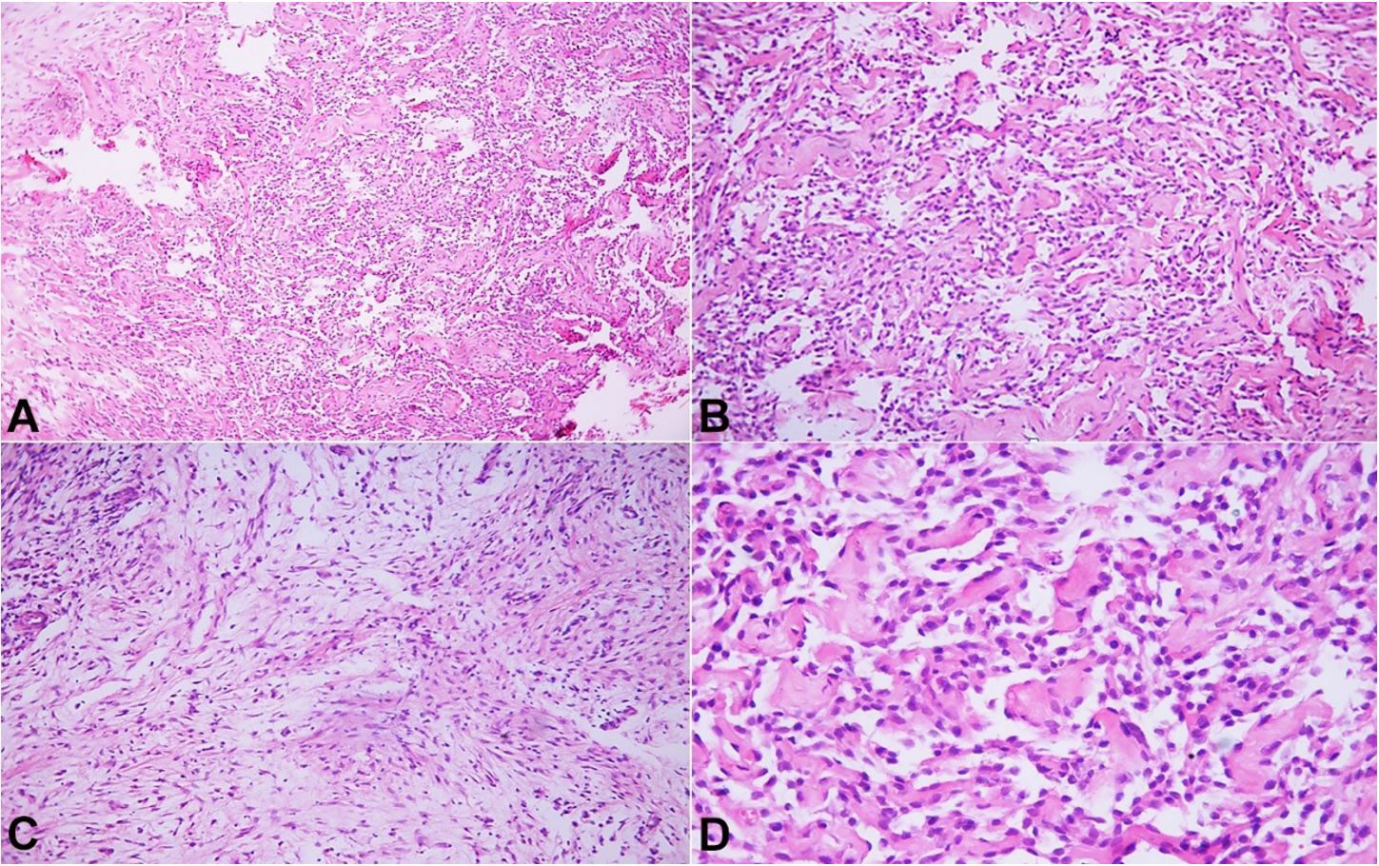
Photomicrograph of the biopsy showed: **A –** biphasic tumor comprising of fibrous as well epithelial component (H&E,10X); **B –** epithelial component disposed mainly in a glandular pattern along with cords (H&E,20X); **C –** shows an area of fibrous component (H&E,20X); **D –** Higher magnification shows round to oval epithelial cells with bland nuclear features and mild to moderate cytoplasm, No pleomorphism (H&E,40X).

A 41-year-old male patient presented with swelling associated with pain of the right leg, which gradually increased over the last two and a half years. There was no previous history of trauma, tuberculosis, or any other significant comorbidity. Overall, the patient’s health status was good except for the leg swelling and associated pain. The clinical examination revealed a firm and tender swelling on the right leg’s anterolateral surface of 20 cm in its longest axis. The overlying skin appeared normal. The remaining systemic examination was normal. All the hematological and biochemical parameters were within the normal range. The X-ray of the leg showed a cortical erosion in the tibia diaphysis and associated soft tissue involvement. The biopsy histomorphology showed a biphasic tumor comprising fibrous stroma with cords and glandular pattern of epithelial cells exhibiting a central discohesion (Figures 1A, 1B). Some areas show fibrous components (Figure 1C). The high-power examination showed round to oval epithelial cells with bland nuclear features and mild to moderate cytoplasm, and no pleomorphism (Figure 1D).

**Figure 2:**
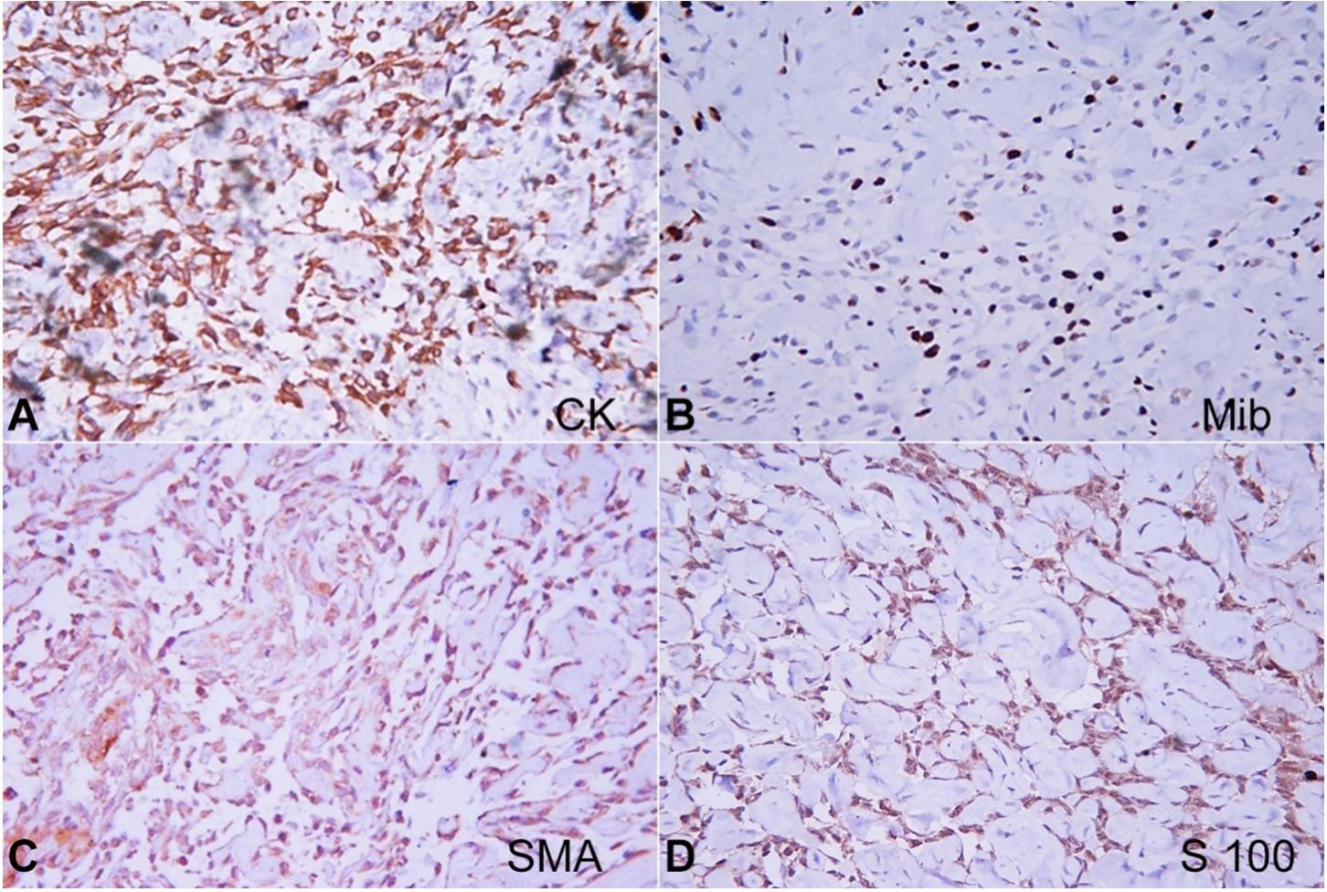
Photomicrographs of the IHC study on the biopsy specimen. **A –** CK positive; **B –** Mib proliferation index 20-30%; **C –** SMA – negative; **D –** S100 equivocal. (all pictures 40X).

The immunohistochemistry showed a Pan CK positivity, MIB proliferation index (KI 67) 20-30%, SMA-negative, S100-equivocal (Figures 2A, 2B, 2C, and 2D). CD34 was negative.

**Figure 3:**
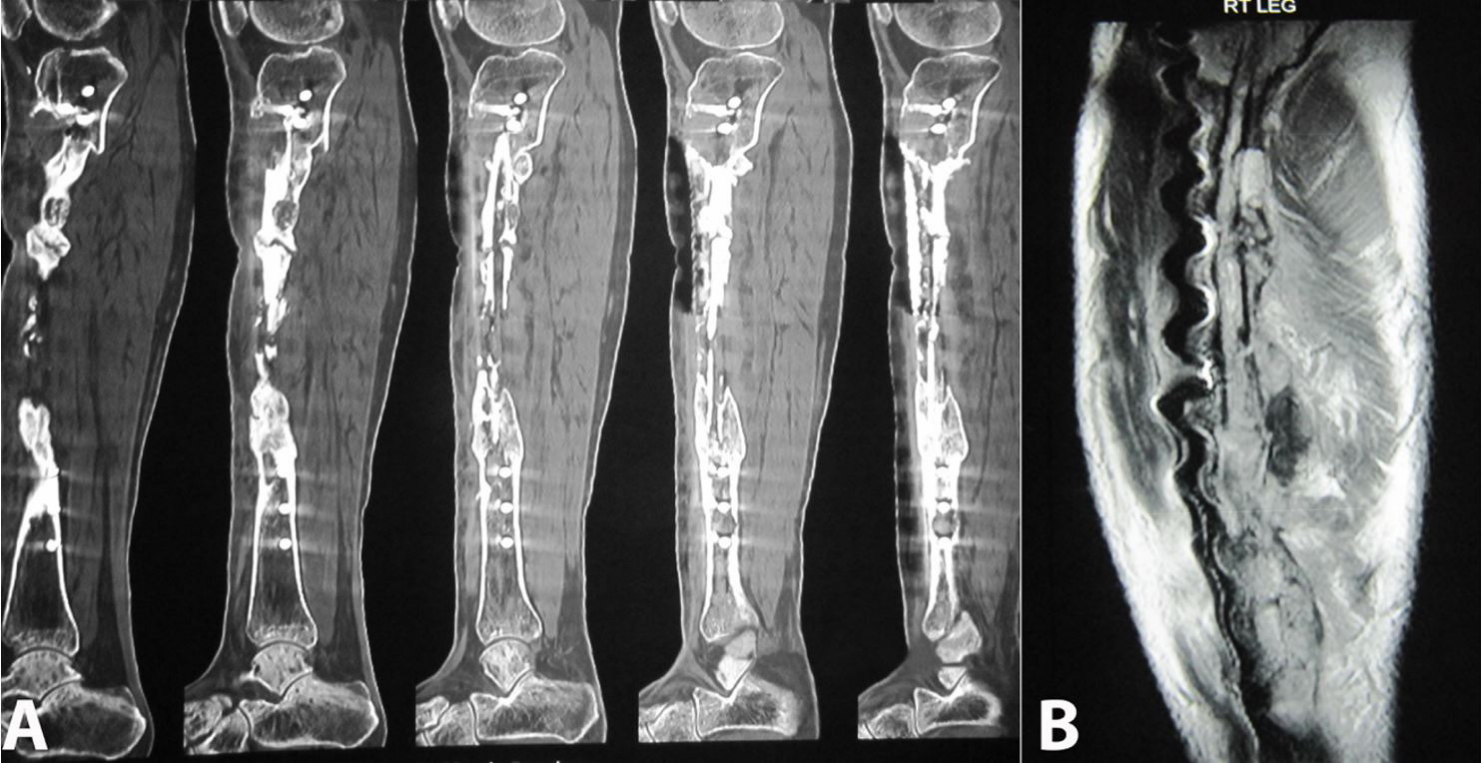
CT study of right leg: **A –** Sagittal sections reveal multiple patchy cortical erosions/irregularities in the right tibia centered in the diaphysis and contiguously involving the adjacent upper metaphysis. Note adjoining soft tissue thickenings. Streak artifacts are noted due to postoperative changes (Screws); **B –** MRI Sagittal section of right leg reveals cortical erosions/irregular outlines of the tibia with altered signals in the adjoining bone marrow and soft tissue. Susceptibility artifacts are noted due to postoperative metallic screws.

The computed tomography (CT) study of the right leg revealed multiple patchy cortical erosions/irregularities in the right tibia in the diaphysis and contiguous involvement of adjacent upper metaphysis. There were adjoining soft tissue thickenings. Streak artifacts are noted due to postoperative changes (screws) (Figure 3A).

The MRI sagittal section of the right leg reveals cortical erosions/ irregular outlines of the tibia with altered signals in the adjoining bone marrow and soft tissue. Susceptibility artifacts are noted due to postoperative metallic screws (Figure 3B). The patient was followed for 3 years, with no signs of metastasis or local relapse.

## DISCUSSION

Adamantinoma is a rare primary low-grade malignant tumor of the bone affecting patients aging 2 years to 86 years, with the median of 25 to 35 years, and with a predominance among women (female: male ratio of 5:4).^14^ ^,^ ^15^ Adamantinoma mostly involves the tibia and fibula; however, other bones can also be affected.

Several histological patterns have been described, such as (i) tubular, (ii) basaloid, (iii) squamous, (iv) spindle-cell, and (v) osteofibrous dysplasia-like variant. Out of these four histological patterns, the basaloid and spindle variants may present a more aggressive behavior. The tubular adamantinoma consists of thin cords of epithelial cells with central discohesion resulting in a vascular or glandular appearance. In the basaloid variant, the epithelial cells exhibit solid nests of basaloid cells with distinctive peripheral palisading. The squamous variant with or without keratinization may resemble well-differentiated squamous cell carcinoma. The spindled form shows uniform spindling with the presence of clefts lined by epithelial cells. The pattern of osteofibrous dysplasia predominantly characterizes the histologic features of the differentiated adamantinoma. Foci of calcification, giant cells, xanthoma, and spindle cells have also been described in adamantinoma. Izquierdo et al.^16^ reported an adamantinoma case with a sarcomatoid transformation that showed a complete loss of epithelial differentiation, and Povýšil et al.^17^ reported a rhabdoid variant of differentiated adamantinoma.

The long bone adamantinomas can be further divided into 2 main groups according to the clinical, radiologic, and histologic data. The first group, defined as classical adamantinoma, is characterized by the abundance of tumor cells forming basaloid, spindled, tubular, or squamoid patterns. In this group, most of the tumor cells strongly stain for cytokeratin. Radiologically, some of these tumors are intracortical, whereas others may expand into the bone medulla, producing large lytic lesions, or toward the soft tissues. This supports the notion that at least some of these tumors are locally aggressive. The patient’s age with classic adamantinoma ranges from 15 to 65 years (mean, 40 years).

The second group is defined as differentiated adamantinoma, which is characterized histologically by a predominance of osteofibrous dysplasia-like pattern with a small inconspicuous component of epithelial elements scattered within the fibroblastic stromal tissue. The epithelial elements consist of small nests, tubular structures, and individual cells that stain positively for cytokeratin. Radiologically, differentiated adamantinomas are uniformly intracortical, producing lytic or sclerotic lesions that are localized predominantly in the anterolateral cortex of the tibia but occasionally in the fibula as well. All patients with this type of adamantinoma are in the first or second decades of life.

The CT scan may demonstrate the soft tissue extension and cortical involvement. CT scan plays a role in the routine work-up of adamantinomas and is also useful in detecting pulmonary metastases. The MRI plays a crucial role in locoregional staging since it depicts distant cortical foci, intramedullary and soft tissue extension. The MRI is also a useful tool for determining tumor-free margins and the strategy for reconstructive surgery.^18^

Two morphological patterns of adamantinoma are described on MRI: (i) multiple small nodules in one or more foci and (ii) solitary lobulated focus. Adamantinomas usually show a low signal intensity on T1-weighted images and a high signal on T2-weighted images. However, these findings are nonspecific.^19^ ^,^ ^20^

It has been postulated that the predominance of the osteofibrous dysplasia-like pattern in differentiated adamantinoma results from a secondary reparative process overgrowing matured and regressing tumor tissue. This process may lead to the total elimination of the recognizable tumor cells from the lesion. Therefore, osteofibrous dysplasia, which has a similar anatomic location, age distribution, and radiologic appearance as differentiated adamantinoma, may, in some cases, represent the evolution of an underlying adamantinoma.

Bishop et al.^21^ reported adamantinoma-like Ewing family tumors of the head and neck. There may be difficulty in making the diagnosis as they display significant histologic overlap with other more common undifferentiated malignancies.

Regardless of the histologic subtypes, all adamantinomas have been uniformly positive for keratins 14 and 19. Immunohistochemically, the epithelial cells show keratin expression, especially basal epithelial cell keratins (CKs 5, 14, and 19) and vimentin. The keratin immunoreactivity pattern is independent of the histologic subtype, despite the marked variety in differentiation pattern, suggesting a common histogenesis for all adamantinoma subtypes.

The cytogenetic analysis has been performed to understand the histopathogenesis of adamantinoma and osteofibrous dysplasia. The trisomies 7, 8, and 12 suggests a common histogenesis of osteofibrous dysplasia and adamantinoma.^22^

Adamantinoma may resemble numerous conditions like an aneurysmal bone cyst, unicameral bone cyst, fibrous dysplasia, chondromyxoid fibroma, giant cell tumor, eosinophilic granuloma, osteomyelitis, chondrosarcoma, hemangioendothelioma, angiosarcoma, and non-ossifying fibromas. Clinical history, site, age, radiological and histopathological features, along with another ancillary testing, can be used to differentiate it from other clinical condition (Table 1).^23^

**Table 1:** Differential diagnosis of Adamantinoma

Lesions	Age (yrs)	Site	Clinical Features	Radiological Features	Gross findings	Microscopy
Aneurysmal bone cyst	10-15	Metaphysis of vertebrae, flat bones, humerus, tibia	Usually, history of trauma, f/b increasing swelling with little pain. There may be pathological fracture or spinal pressure symptoms	Well defined radiolucent, eccentric cyst	Spongy hemorrhagic mass	Fibrous tissue, vascular spaces
Unicameral bone cyst	10-20	Metaphysis of Humerus, femur	Usually, asymptomatic	Well demarcated, radiolucent cyst extending up to physeal plate	Cystic mass	Well vascularized fibrous tissue with hemosiderin and cholesterol clefts
Fibrous dysplasia	10-30	Metaphysis, diaphysis of Neck of femur, tibia, base of skull	May be mono or polyostotic, Pathological fractures and progressive deformity	Cystic areas in metaphysis, lucent patches typically have ground glass appearance	Coarse gritty, Grayish yellow	Loose cellular fibrous tissue with widespread patches of woven bone and scattered giant cells
Chondromyxoid Fibroma	10-25	Metaphysis of tibia, fibula, femur, feet, pelvis	Asymptomatic, pathological fracture	Eccentrically placed lytic lesion with well-defined sclerotic margins	Solid yellowish white or tan	Patches of myxomatous tissue with stellate cells, islands of hyaline cartilage, fibrous tissue
Giant cell tumor	20-40	Epiphysis and metaphysis of Femur, tibia, radius	Pain with swelling, pathological fracture	Eccentric, cystic lesion in mature bone, extending up to the subchondral plate, soap bubble appearance	Reddish fleshy mass	Multinucleated giant cells, stromal cells, cellular atypia with mitotic figures
Eosinophilic granuloma	05-10	Metaphysis of Flat bones, mandible, spine and long bones	Local pain, swelling and tenderness	Well demarcated oval radiolucent area, associated with marked reactive sclerosis	Soft, granular or gelatinous mass	Sheets of Langerhans cells
Osteomyelitis	Any age	Metaphysis, diaphysis of femur, tibia, femur, and humerus	Discharging sinus, fever, malaise, local pain and swelling	Multiple aggressive lytic lesions, serpiginous lytic pattern is more Specific sequestrum and involucrum are often seen	Bone destruction, cavities containing pus with sequestrum	Inflammatory cells around areas of acellular bone or microscopic sequestrum, prominent periosteal bone proliferation
Chondro-sarcoma	30-60	Pelvis rib, vertebrae, and metaphysis of humerus, femur	Dull ache or gradually enlarging lump	Radiolucent area with central flecks of calcification	Lobulated with gelatinous shiny areas	Lobules of highly atypical cells, including binucleate cells
Epithelial metastasis	Any age	Diffuse pattern of Vertebrae, pelvis, rib, femur, skull, humerus rare below elbow and knees)	Pain	Bone destruction, osteolytic; osteoblastic response with Ca prostate	Osteolytic, rarely sclerotic	Malignant cells with vascular invasion
Hemangio-endothelioma	20-30	Metaphysis, diaphysis, less commonly, epiphysis of femur, tibia, feet and calvarium	Pain and swelling	Expansive, osteolytic and poorly demarcated lesions. “soap bubble” matrix with a sclerotic margin	Well-circumscribed, irregular borders soft, bright red hemorrhagic appearance	Solid nests and anastomosing cords of round, polygonal, or spindle-shaped cells with eosinophilic cytoplasm. Intracytoplasmic vacuolization
Angiosarcoma	Any age	Metaphyseal and diaphyseal of any bone, multifocal	Pain and swelling	Eccentric, lytic, metaphyseal and diaphyseal, well circumscribed areas of rarefaction	Variable	Anastomosing vascular channels lined by highly atypical endothelial cells
Non ossifying fibromas	10-20	Metaphysis of tibia, femur	Pain	Eccentric, sharply delimited lesion	Solid, Granular, brown, dark red	Fibrous tissue arranged in storiform pattern, foamy and hemosiderin laden macrophages

Adamantinomas are locally aggressive tumors and are extremely slow-growing with the potential to metastasize. Prognosis is excellent if excised early with a wide margin. Metastasis to the lung or lymph nodes is as high as 12-29%.^23^ Recurrence of the tumor is frequent after inadequate therapy, and the behavior of the recurrent neoplasm resembles more and more a sarcoma. The local recurrence rate ranges between 18 and 32%. However, the precise 5-year mortality rate is difficult to ascertain because of the rarity of this tumor.

The current adamantinoma treatment includes *en bloc* tumor resection with wide operative margins, limb reconstruction, and limb salvage. This approach provides lower rates of local recurrence than the previously reported data.^23^ Unfortunately, neither radiation therapy nor chemotherapy has shown effectiveness in the treatment of this insidious tumor.

## CONCLUSION

Although the adamantinoma incidence is low, it is important to recognize this rare bone tumor since there is a possibility of recurrence, as well as metastasis if not adequately managed. The histologic features of primary adamantinoma are usually characteristic enough for a presumptive diagnosis; however, the rarity and the heterogeneity of the tumor could pose diagnostic uncertainty in some cases, especially those arising in non-tibial locations. Extensive sampling of the lesion is important, especially in the differentiated adamantinoma, where the epithelial component may only be seen focally. To assure the histological diagnosis Pathologists should employ immunohistochemistry for demonstrating the eventual sparse epithelial cell nests when radiology is suggestive for adamantinoma. Correct diagnosis should lead to resection with wide surgical margins.
